# Enhanced Field‐Like Torque Generated from the Anisotropic Spin‐Split Effect in Triple‐Domain RuO_2_ for Energy‐Efficient Spin–Orbit Torque Magnetic Random‐Access Memory

**DOI:** 10.1002/advs.202413165

**Published:** 2025-02-28

**Authors:** Thi Van Anh Nguyen, Hiroshi Naganuma, Thi Ngoc Huyen Vu, Samik DuttaGupta, Yoshiaki Saito, Duong Vu, Yasushi Endo, Shoji Ikeda, Tetsuo Endoh

**Affiliations:** ^1^ Center for Science and Innovation in Spintronics (Core Research Cluster) Tohoku University Katahira 2‐1‐1, Aoba ku Sendai Miyagi 980‐0812 Japan; ^2^ Center for Innovative Integrated Electronic Systems Tohoku University 468‐1 Aramaki‐Aza Aoba, Aoba‐ku Sendai Miyagi 980‐8572 Japan; ^3^ Institute for Advanced Study Nagoya University Furo‐cho, Chikusa‐ku Nagoya Aichi 464‐8601 Japan; ^4^ Institute of Materials and Systems for Sustainability Nagoya University Furocho, Chikusa‐ku Nagoya Aichi 464‐8601 Japan; ^5^ Institute for Materials Research Tohoku University 2‐1‐1 Katahira, Aoba‐ku Sendai Miyagi 980‐8577 Japan; ^6^ Saha Institute of Nuclear Physics Sector‐1, Block‐AF, Bidhan nagar, Kolkata West Bengal West Bengal 700 064 India; ^7^ Research Institute of Electrical Communication Tohoku University 2‐1‐1 Katahira, Aoba‐ku Sendai Miyagi 980‐8577 Japan; ^8^ Institute of Physics Vietnam Academy of Science and Technology 10 Dao Tan, Ba Dinh Hanoi Hanoi Vietnam; ^9^ Graduate School of Engineering Tohoku University 6‐6, Aramaki Aza Aoba, Aoba‐ku Sendai Miyagi 980‐8579 Japan

**Keywords:** altermagnet RuO_2_, field‐like torque, magnetic random‐access memory, spin‐orbit torque, spin‐split effect

## Abstract

Spin‐current generation via the anisotropic spin‐split effect has been predicted in antiferromagnetic RuO_2_, where the symmetry of RuO_2_ plays a critical role in spin–orbit torque (SOT). This phenomenon has garnered attention for its potential to enable energy‐efficient spintronic devices, such as SOT magnetic random‐access memory. In this study, a high‐quality RuO_2_ (100) epitaxial film with a well‐controlled triple‐domain‐structure is analyzed, and it is confirmed that out‐of‐plane spin‐current generation is independent of the Néel vector ($\vec{N}$). This $\vec{N}$ independence of the out‐of‐plane spin current leads to equal SOT values for the two orthogonal currents. The spin‐split effect‐induced SOT demonstrates a field‐like (FL) torque efficiency (−0.066 ± 0.001) that is six times higher than that of the Slonczewski‐like torque efficiency (−0.011 ± 0.001). Furthermore, micromagnetic simulations show that this high FL torque reduces the critical switching voltage by a factor of 2.6 in the sub‐nanosecond regime in an SOT device. These findings contribute to advancing research and the development of highly energy‐efficient antiferromagnetic‐based SOT magnetic random‐access memory.

## Graphical abstract

This study shows an unexplored avenue toward engineering spin‐split effect‐induced spin‐orbit torque (SOT) in triple‐domain structured (100) RuO_2_/Co‐Fe‐B system where the field‐like torque is significantly enhanced compared with the Slonzewsky‐like torque. Thanks to this achievement, a high energy‐efficient antiferromagnetic‐based spin‐orbit torque (SOT) magnetic random‐access memory will be expected as demonstrated using the micro‐magnetic simulation.

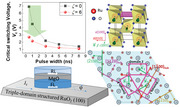

## 1 Introduction

The generation of a spin current (*J*_S_) by a charge current (*J*_C_) is a key phenomenon in spintronics research. While longitudinal spin‐polarized currents with odd behavior under time reversal (T_odd) have been used in commercialized spin‐transfer torque magnetic random‐access memory (STT‐MRAM),^[^ ^1^, ^2^, ^3^ ^]^ transverse spin‐polarized currents with even behavior under time reversal (T_even) have been investigated in spin–orbit torque magnetic random‐access memory (SOT‐MRAM).^[^ ^4^, ^5^, ^6^, ^7^, ^8^ ^]^ The spin–orbit torque (SOT) observed in nonmagnetic (NM)/ferromagnetic (FM) bilayers comprises two orthogonal components:^[^ ^9^, ^10^ ^]^ the Slonczewski‐like and field‐like (SL and FL, respectively) torques, which mainly originate from the conventional spin Hall effect (SHE)^[^ ^5^ ^]^ and/or interfacial Rashba effect.^[^ ^11^, ^12^ ^]^ Despite the debate over their origins, SOT is appealing for MRAM applications owing to its significant advantages, such as ultrafast write speeds and high endurance.^[^ ^6^, ^7^, ^8^ ^]^ An ultra‐fast write speed of 350 ps in SOT‐MRAM utilizing a canted SOT structure has been demonstrated,^[^ ^6^ ^]^ expanding the potential for spintronic applications, such as static random‐access memory and/or caches replacement. To realize SOT‐MRAM applications, higher energy efficiency is required, which can be achieved by tailoring the physical parameters of the stack, such as the magnetic anisotropy and ratio between the FL and DL torques, known as the FL coefficient (*ζ*).^[^ ^13^ ^]^ Thus, the material engineering of the NM/FM stack structure is essential for realizing efficient SOT‐MRAM applications.

**Figure 1:**
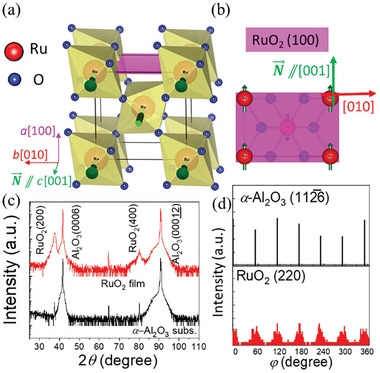
a) Crystal structure of rutile‐type RuO_2_. b) RuO_2_ (100) plane with the Néel vector $\left(\vec{N}\right)$ aligned along the [001] direction. c) X‐ray diffraction *θ‐* and d) *φ*‐scans of the $\left(11\bar{2}6\right)$ diffraction plane of the *α*‐Al_2_O_3_ (0001) substrate and (220) diffraction planes of the RuO_2_ (100) film.

Heavy metals are commonly used in NM layers, where the T_even spin current is generated through the SHE. However, special materials such as RuO_2_, which possess Dirac nodal lines protected by nonsymmorphic symmetries in their band structure, have also induced T_even spin currents via the SHE.^[^ ^14^ ^]^ Recently, a distinct mechanism for generating T_odd spin currents generation from nonrelativistic collinear antiferromagnets was predicted, with the contribution of an anisotropic spin‐split effect (SSE).^[^ ^15^, ^16^ ^]^ Among the various antiferromagnets applicable to this mechanism, rutile RuO_2_ is a representative candidate due to its room‐temperature antiferromagnetic and metallic properties.^[^ ^17^ ^]^ Antiferromagnetism in RuO_2_ occurs when the rutile symmetry (*P*4_2_/*mnm*) is distorted, causing the Néel vector ($\vec{N}$) to align along the [001] axis.^[^ ^17^, ^18^ ^]^ The rutile structure of collinear antiferromagnetic RuO_2_, shown in **Figure** **1**a, consists of Ru atoms with opposite spins, which are surrounded by different crystal fields of oxygen octahedrons (90° rotation). Figure 1b illustrates the RuO_2_ (100) plane with the $\vec{N}$ aligned parallel to the [001] direction. This collinear antiferromagnetic structure enables the formation of an anisotropic spin‐split band in momentum space when a ${\vec{J}}_{C}$ is applied along a specific direction to the RuO_2_ crystal, resulting in SSE‐induced spin‐current generation with high efficiency.^[^ ^15^ ^]^ This implies that the generation of SSE‐induced spin currents is highly dependent on the directions of both the ${\vec{J}}_{C}$, and $\vec{N}$.

Because of the anisotropic nature of the SSE, the experimentally observed SSE‐induced spin‐current generation varies depending on the crystal structures of RuO_2_. For example, SSE‐induced spin‐current generation in single‐crystal RuO_2_ (101) has been reported,^[^ ^19^ ^]^ with a complex $\vec{N}$ dependence of the spin Hall conductivity (SHC) on the $\vec{N}$, which was attributed to its canted orientation in RuO_2_ (101). As a result, the angle‐dependent SOT was measured, revealing an SL component (7  ×  10^3^ (Ωm)^−1^), whereas the FL component was negligible.^[^ ^19^ ^]^ In contrast, another study experimentally observed SSE‐induced spin‐current generation in RuO_2_ (100)^[^ ^20^ ^]^ and evaluated the SOT efficiency per unit electric field at 4  ×  10^4^ (Ωm)^−1^. However, this study did not account for the effects of multidomain structures, which could influence the SOT evaluation.^[^ ^21^ ^]^ Therefore, to achieve high‐energy‐efficient SOT‐MRAM, precise evaluation of the SSE‐induced SOT is crucial, requiring thorough investigation of the crystal structure and symmetry analysis of the SHC.

In this study, a RuO_2_ (100) epitaxial film with a well‐controlled triple‐domain structure was fabricated, in which each domain was rotated by 120° relative to the others, resulting in a corresponding 120° rotation of the $\vec{N}$. The SSE‐induced SOT in a RuO_2_/Co_20_Fe_60_B_20_ bilayer was investigated using harmonic Hall measurements, and the experimental data was explained by calculating the SHC for the triple‐domain structure through symmetry analysis of RuO_2_ (100) with $\vec{N}$//[001]. The SHC was confirmed to be independent of the $\vec{N}$, allowing for precise evaluation of the SL and FL torques with a significantly high *ζ*. Furthermore, micromagnetic simulations demonstrated the benefit of the high *ζ* in reducing the switching voltage in a canted SOT device, offering the potential for high‐energy‐efficient SOT‐MRAM applications.

## 2 Experimental Procedures

A RuO_2_ (4)/Co_20_Fe_60_B_20_ (1.2)/MgO (1.3)/Ta (1.0) stack film, hereafter referred to as RuO_2_/CFB, was fabricated on *α*‐Al_2_O_3_ (0001) using DC and radiofrequency sputtering, with the numbers in parentheses indicating the nominal thickness in nanometers. Detailed sample preparation is provided in Section S1 (Supporting Information), and the formation of RuO_2_ is confirmed through X‐ray absorption spectra in Section S2 (Supporting Information). X‐ray diffraction (XRD) with Cu *K*α radiation (Bruker AXS, D8 Discover) was conducted to characterize the crystal quality of the films. Both *θ‐* and *φ*‐scans were used to analyze the crystal structure and epitaxial relationship of the RuO_2_ film on *α*‐Al_2_O_3_ (0001). The SHC of the RuO_2_ (100) film was calculated based on symmetry analysis, as described in previous studies.^[^ ^15^, ^19^ ^]^ The SOT was evaluated through harmonic Hall resistance measurements.

## 3 Results and Discussion

**Figure 2:**
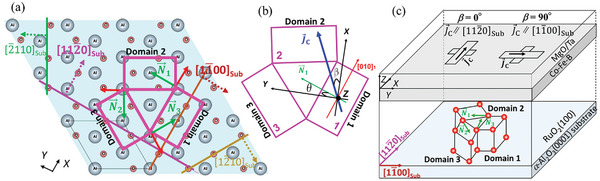
a) Illustration of the triple‐domain structure formation of RuO_2_ (100) on the *α*‐Al_2_O_3_ (0001) substrate, generated using VESTA,^[^ ^31^ ^]^ with each crystal axis represented by dashed‐line arrows. b) Schematic of the applied charge current (${\vec{J}}_{C}$) at an angle *β* relative to the *X*‐axis, where [010]_1_ denotes the [010] axis of Domain 1. c) Illustration of the Hall bar devices fabricated on the stacked structure.

Figure 1c,d shows the XRD *θ‐* and *φ‐*scans of a RuO_2_ film grown on an *α*‐Al_2_O_3_ (0001) substrate. The clear peaks corresponding to RuO_2_ (200) and (400) confirmed the formation of a (100)‐oriented RuO_2_ film on the substrate. The XRD *φ‐*scan in Figure 1d shows the six‐fold symmetry of the RuO_2_ film in the in‐plane direction, as indicated by the (220) peaks of RuO_2_ and (11$\bar{2}$6) peaks of *α*‐Al_2_O_3_. This result suggested the formation of a triple‐domain‐structure with the epitaxial relationship: RuO_2_[010]//*α*‐Al_2_O_3_[11$\bar{2}$0], [$\bar{2}$110], and [1$\bar{2}$10].^[^ ^22^, ^23^ ^]^ **Figure** **2**a illustrates the formation of this triple‐domain structure, in which the Néel vector of each domain, ${\vec{N}}_{i}$, indexed by *i*, is rotated by 120°.

The SHC for a RuO_2_ (100) film with a well‐controlled triple‐domain structure was calculated based on symmetry analysis.^[^ ^15^, ^19^, ^24^ ^]^ The SHC is described by tensors with different components, with non‐zero components including $\sigma_{yz}^{x}$, $\sigma_{zy}^{x}$, $\sigma_{zx}^{y}$, $\sigma_{xz}^{y}$, $\sigma_{xy}^{z}$, and $\sigma_{yx}^{z}$ (Section S3, Supporting Information). These components denote the directions of the spin current, spin polarization, and *J_C_*. For example, $\sigma_{zy}^{x}$ refers to the component where the spin polarization is in the *x‐*direction, the spin current flows in the *z*‐direction, and the applied *J_C_* is in the *y‐*direction. The magnitude of this component is given by $\sigma_{zy}^{x}=-C\sin^{2}\theta +A\cos^{2}\theta$, where *C* and *A* are the components of the SHC tensors, as reported in the previous study.^[^ ^19^ ^]^

If θ represents the angle between the *Y*‐axis and $\vec{N}$ of Domain 1 (${\vec{N}}_{1}$) (Figure 2b), then the angles between the *Y*‐axis and $\vec{N}$ values of Domains 2 and 3 (${\vec{N}}_{2}$ and ${\vec{N}}_{3}$) are (θ + 120) and (θ + 240)°, respectively. Subsequently, the SHC components for the triple‐domain system were calculated using these reindentation's. For example, the equation for $\sigma_{zy}^{x}$ is given by:

(1) $$\begin{array}{ccc} & & \sigma_{\mathrm{zy}}^{x}=\sigma_{\mathrm{zy}}^{x,\mathrm{Domain}\;1}+\sigma_{\mathrm{zy}}^{x,\mathrm{Domain}\;2}+\sigma_{\mathrm{zy}}^{x,\mathrm{Domain}\;3}\sigma_{\mathrm{zy}}^{x}\\ & & =-C[\sin^{2}\theta +\sin^{2}\left(\theta +120\right)+\sin^{2}\left(\theta +240\right)]\\ & & +A\left[\cos^{2}\theta +\cos^{2}\left(\theta +120\right)+\cos^{2}\left(\theta +240\right)\right]=\frac{3}{2}\left(A-C\right) \end{array}$$

Equation (1) shows that $\sigma_{zy}^{x}$ does not depend on θ, and similarly, all components of the SHC tensors in the triple‐domain structure are independent of θ. This implies that the total SHC is independent of the $\vec{N}$ orientation owing to the triple‐domain structure. A potential concern could be the orientation of the $\vec{N}$ along the [00$\bar{1}$] direction, which is 180° rotated from the [001] direction. However, this does not affect the SHC evaluation using Equation (1) because the SHC for each domain depends on θ through sin ^2^θ and cos ^2^θ.

Next, we define β as the angle between the ${\vec{J}}_{C}$ and *X*‐axis (Figure 2b). The ${\vec{J}}_{C}$ has two components along the *X‐* and *Y*‐axes: $J_{C\_X}=J_{C}\;\cos\beta$ and $J_{C\_Y}=J_{C}\;\sin\beta$. Therefore, the spin currents along the *X*‐, *Y*‐, and *Z*‐axes can be expressed as

(2) $$J_{S\_X}^{Z}\;∼\;\sigma_{xy}^{z}J_{C}\sin\beta =\frac{3}{2}\left(A-B\right)\sin\beta J_{C}$$

(3) $$J_{S\_Y}^{Z}\;∼\;\sigma_{yx}^{z}J_{C}\cos\beta =\frac{3}{2}\left(B-A\right)\cos\beta J_{C}$$

(4) $$\begin{array}{ccc} & & J_{S\_Z}^{\mathrm{tot}}=J_{S\_Z}^{X}+J_{S\_Z}^{Y}\;∼\;\left(\sigma_{zy}^{x}\sin\beta +\sigma_{zx}^{y}\cos\beta \right)J_{C}\\ & & =\frac{3}{2}\left(A-C\right)\sin\beta J_{C}+\frac{3}{2}\left(C-A\right)\cos\beta J_{C} \end{array}$$

where *A, B*, and *C* are components of the SHC tensors, as described in a previous report^[^ ^19^ ^]^ (Section S3, Supporting Information). The spin currents in *X‐* and *Y*‐directions are polarized in the *Z*‐direction (Equations (2) and (3)), while the total spin current in the *Z*‐direction (out‐of‐plane spin current) in (Equation (4)) is polarized in the *X‐* and *Y*‐directions (first and second terms in Equation (4), respectively), corresponding to in‐plane polarization.

Since the total SHC does not depend on θ, we set θ  =  0, and β is the angle between the ${\vec{J}}_{C}$ and [010] axis of Domain 1.

If ${\vec{J}}_{C}//{\vec{N}}_{1}$ (β  = 90° ), then:

(5) $$J_{S\_X}^{Z}=\frac{3}{2}\left(A-B\right)J_{C}$$

(6) $$J_{S\_Y}^{Z}=0$$

(7) $$J_{S\_Z}^{\mathrm{tot}}∼\frac{3}{2}\left(A-C\right)J_{C}$$

If ${\vec{J}}_{C}⊥{\vec{N}}_{1}$ (β  = 0° ), then:

(8) $$J_{S\_X}^{Z}=0$$

(9) $$J_{S\_Y}^{Z}=\frac{3}{2}\left(B-A\right)J_{C}$$

(10) $$J_{S\_Z}^{\mathrm{tot}}∼\frac{3}{2}\left(C-A\right)J_{C}$$

Equations (7) and (10) show that the absolute values of $J_{S\_Z}^{\mathrm{tot}}$ are equal for ${\vec{J}}_{C}//{\vec{N}}_{1}$ and ${\vec{J}}_{C}⊥{\vec{N}}_{1}$, suggesting that the SOTs induced by these spin currents are similar. This hypothesis is investigated experimentally in the following section.

To test this suggestion, the SOT of Hall bar devices were evaluated with two orthogonal *J_C_* directions (Figure 2c). Two types of Hall bar devices were fabricated using RuO_2_/CFB films. In the first device, ${\vec{J}}_{C}\;\;{\left.[1\bar{1}00\right]}_{\mathrm{Sub}}$, i.e., ${\vec{J}}_{C}\;\;{\vec{N}}_{1}$ or *β* = 90°. In the second device, ${\vec{J}}_{C}\;\;{\left.[11\bar{2}0\right]}_{\mathrm{Sub}}$, i.e., ${\vec{J}}_{C}\;\;{\vec{N}}_{1}$ or *β* = 0°. An out‐of‐plane spin current with in‐plane polarization was detected using harmonic Hall measurements, where an external magnetic field (*H*_ext_) with a constant magnitude was rotated in the azimuthal plane, and *φ* is the angle between the *H*_ext_ and ${\vec{J}}_{C}$. Based on the harmonic Hall resistance, the SL (*H*_SL_) and FL (*H*_FL_) components of the SOT‐induced effective fields were quantified, excluding any thermal effects.^[^ ^25^ ^]^

**Figure 3:**
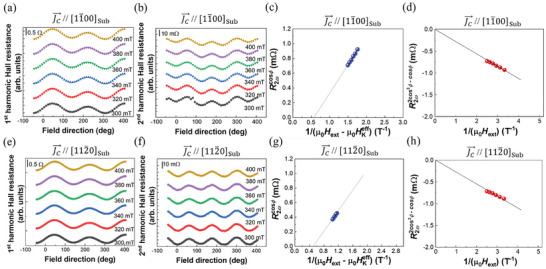
First and second harmonic Hall resistances measured under various external fields for devices with ${\vec{J}}_{C}\;\;{\left.[1\bar{1}00\right]}_{\mathrm{Sub}}$ ((a) and (b)) and ${\vec{J}}_{C}\;\;{\left.[11\bar{2}0\right]}_{\mathrm{Sub}}$ ((e) and (f)). First and second terms of Equation (11) for devices with ${\vec{J}}_{C}\;\;{\left.[1\bar{1}00\right]}_{\mathrm{Sub}}$ ((c) and (d)) and ${\vec{J}}_{C}\;\;{\left.[11\bar{2}0\right]}_{\mathrm{Sub}}$ ((g) and (h)).

The first (*R*_ω_) and second (*R*_2ω_) harmonic Hall resistances were measured using a lock‐in technique for devices with ${\vec{J}}_{C}\;\;{\left.[1\bar{1}00\right]}_{\mathrm{Sub}}$ (**Figure** **3**a,b) and ${\vec{J}}_{C}\;\;{\left.[11\bar{2}0\right]}_{\mathrm{Sub}}$ (Figure 3e,f). The *H*_SL_ and *H*_FL_ were obtained by fitting the *R*_ω_ and *R*_2ω_ versus *φ*, respectively. Prior to the harmonic Hall measurements, the anomalous Hall resistance coefficient (*R*_AHE_) and effective anisotropy field ($H_{K}^{\mathrm{eff}}$) of the devices (Section S4, Supporting Information) were determined, which were necessary for determining the *H*_SL_ and *H*_FL_. The *J*_C_ was evaluated based on the resistivity of each layer (ρ_RuO2_ =  247.4 µΩcm, ρ_CFB_ = 162.7 µΩcm) (Section S5, Supporting Information).

The *R*_1ω _signal is generated by the planar Hall resistance, and the *R*_1ω _versus *φ* curve can be expressed as follows:^[^ ^25^, ^26^, ^27^, ^28^ ^]^ *R*_1ω_ = *R*_PHE_ sin 2φ, where *R*_PHE_ is the planar Hall resistance coefficient, which is influenced by the in‐plane easy‐axis and negligible anisotropy in the film plane.

The *R*_2ω_ signal includes contributions from the Oersted field (*H*_OE_) related to the current applied to the device and SOT effective fields (*H*_SL_ and *H*_FL_). The *R*_2ω _versus *φ* curve is given by:

(11) $$\begin{array}{ccc} & & R_{2\omega }=-\left(R_{\mathrm{AHE}}\frac{H_{\mathrm{SL}}}{H_{\mathrm{ext}}-H_{K}^{\mathrm{eff}}}+R_{T}^{0}\right)\cos\varphi \\ & & +2R_{\mathrm{PHE}}\left(2\cos^{3}\varphi -\cos\varphi \right)\frac{H_{\mathrm{FL}}+H_{\mathrm{OE}}}{H_{\mathrm{ext}}} \end{array}$$

where $R_{T}^{0}$ is the second‐harmonic Hall coefficient originating from thermoelectric effects. The first term corresponds to the cos*φ* contribution of the *R*_2ω,_ expressed as a function of 1/(*µ*_0_ *H*_ext_–*µ*_0_ $H_{K}^{\mathrm{eff}}$), which enables the estimation of the *H*_SL_. The second term corresponds to the (2cos^3^ *φ* – cos*φ*) contribution of the *R*_2ω,_ expressed as a function of 1/(*µ*_0_ *H*_ext_) and facilitates the estimation of the *H*_FL_ after subtracting the contribution of the *H*_OE_ (Section S6, Supporting Information). Linear fitting of these data points enabled the evaluation of the *H*_SL_ and *H*_FL_ (shown as lines in Figure 3c,d,g,h). For each device, we evaluated the *R*_AHE_ and $H_{K}^{\mathrm{eff}}$ (Section S4, Supporting Information) to determine the exact values of the *H*_SL_ and *H*_FL_.

**Figure 4:**
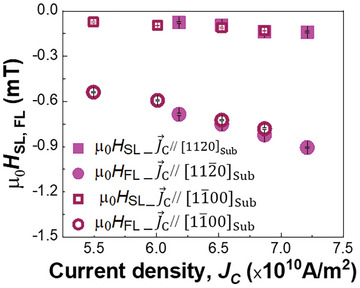
*J*_C_ dependence of *H*_SL_ and *H*_FL_ for devices with ${\vec{J}}_{C}\;\;{\left.[1\bar{1}00\right]}_{\mathrm{Sub}}$ and ${\vec{J}}_{C}\;\;{\left.[11\bar{2}0\right]}_{\mathrm{Sub}}$.

**Figure** **4** shows the *J*_C_ dependence of the *H*_SL_ and *H*_FL_ for devices with ${\vec{J}}_{C}\;\;{\left.[1\bar{1}00\right]}_{\mathrm{Sub}}$ (*β* = 90°) and ${\vec{J}}_{C}\;\;{\left.[11\bar{2}0\right]}_{\mathrm{Sub}}$ (*β* = 0°), represented by open and closed symbols, respectively. Both |*H*_SL_| and |*H*_FL_| increase with *J*_C_, implying a current‐induced origin for the observed behavior. Moreover, we obtained comparable magnitudes of |*H*_SL_| and |*H*_FL_| for both devices at similar applied current densities. This result aligns with the symmetry analysis of the SHC from the previous section. A potential concern might be the SSE origin of the observed results, as similar values of SOT fields would be expected if no anisotropic SSE were present in the RuO_2_ film. To address this, we fabricated control devices patterned on a (110)‐RuO_2_ (5)/Co_20_Fe_60_B_20_(2)/MgO(1.3)/Ta(1) film stacked on a TiO_2_ (110) substrate. The results from harmonic Hall measurements show no SSE‐induced SOT in this device, as no out‐of‐plane spin current is generated in RuO_2_ (110)^[^ ^15^ ^]^ (Section S7, Supporting Information). In addition, we fabricated a device with *β* = 45° and evaluated its effective SOT fields (Section S8, Supporting Information) for comparison with those of devices having *β* = 0° and *β* = 90°. The results show that the SOT effective fields for these devices are similar, which is consistent with the calculation of the out‐of‐plane spin current in Equation (4), based on the SSE‐induced SOT phenomenon in the triple‐domain‐structured (100) RuO_2_/CFB bilayer. This implies that the observed SOT in this system can be attributed to the $\vec{N}$‐independent features of the SSE.

The SOT efficiencies (*ξ*_SL_ and *ξ*_FL_) were estimated following the relationship: $\xi_{\mathrm{SL}(\mathrm{FL})}=\frac{2eH_{\mathrm{SL}(\mathrm{FL})}m_{S}}{\hbar J_{C}}\;,$ where *e* is the electron charge, ℏ is the Dirac constant, *m*_S_ is the areal magnetic moment. At *J*_C_ = 6.87 × 10^10^ A m^−2^, the efficiencies are ξ_SL_ = −0.011 ± 0.001 and ξ_FL_ = −0.066 ± 0.001. We also evaluated the SL/FL‐induced SOT efficiency per unit electric field, ξ_SL,E_ and ξ_FL,E_, following the equation: $\xi_{\mathrm{SL}(\mathrm{FL}),E}=\frac{\hbar }{2e}\;\frac{\xi_{\mathrm{SL}(\mathrm{FL})}}{\rho_{\mathrm{RuO}2}}$, where ρ_RuO2_ is the longitudinal resistivity of RuO_2_ (100). The results are summarized in **Table** **1** in comparison with previous studies.^[^ ^18^, ^19^ ^]^

**Table 1:** Summary of SOT efficiencies per unit electric field for various RuO_2_ films.

Structure	Characterization method	SHE depends on		ξ_SL,E_ (Ωm)^−1^	ξ_FL,E_ (Ωm)^−1^
		$\vec{N}$	${\vec{J}}_{C}$		
RuO_2_ (101)/TiO_2_ (101) substrate (Single domain)^[^ ^18^ ^]^	ST‐FMR & Harmonic Hall Measurement	Yes	Yes	7 × 10^3^	–
RuO_2_ (100)/YSZ (100) substrate (Multi domain)^[^ ^19^ ^]^	ST‐FMR	Yes	Yes	4 × 10^4^	–
RuO_2_ (100)/*α*‐Al_2_O_3_ (0001) substrate (Triple domain) [Present study]	Harmonic Hall Measurement	No^a)^	Yes	4.4 × 10^3^	2.7 × 10^4^

^a)^ Owing to the triple‐domain structure. $\vec{N}$ and ${\vec{J}}_{C}$ represent the orientation of the Néel vector and direction of the applied current, respectively. Abbreviations: SHE, spin Hall effect; SOT, spin–orbit torque, ST‐FMR: Spin torque ferromagnetic resonance.

The evaluated ξ_SL,E_ = (4.4  ±  0.3)  ×  10^3^(Ωm)^−1^ is comparable to that of RuO_2_ (101) reported elsewhere,^[^ ^19^ ^]^ while ξ_FL,E_ = (2.7  ±  0.1)  ×  10^4^(Ωm)^−1^ is similar to the SOT of Pt (≈0.07).^[^ ^29^, ^30^ ^]^ Notably, ξ_FL,E_ is six times larger than ξ_SL,E_, indicating that the FL torque coefficient (*ζ*) is 6 for the triple‐domain structured RuO_2_ (100)/Co–Fe–B system. Such material, with a high *ζ*, holds promise for realizing ultralow‐power‐consuming SOT‐MRAM applications, as demonstrated in our previous report.^[^ ^13^ ^]^ It should be noted that the crystallinity of the RuO_2_ film is determined by the substrate and growth conditions.^[^ ^19^, ^20^, ^21^, ^22^, ^23^ ^]^ Variations in the crystallinity lead to changes in the SHC of the RuO_2_ film, as well as the SSE‐induced SOT in the RuO_2_/FM bilayer.^[^ ^15^, ^19^, ^20^, ^21^ ^]^ While a direct comparison between the RuO_2_ (100) crystal structure in this study (with its triple‐domain structure) and that of a previous report^[^ ^20^ ^]^ is challenging, the use of the triple‐domain structure in RuO_2_ (100) grown on the *α*‐Al_2_O_3_ (0001) substrate (Fig. 2 (a) [31]) allowed for simplification of the angle dependence feature of the SHC. This approach enabled the precise evaluation of both of ξ_SL,E_ and ξ_FL,E_ values in our experiments. A higher SOT efficiency could be achieved by further optimizing the crystal quality, as suggested in a previous study.^[^ ^15^ ^]^

**Figure 5:**
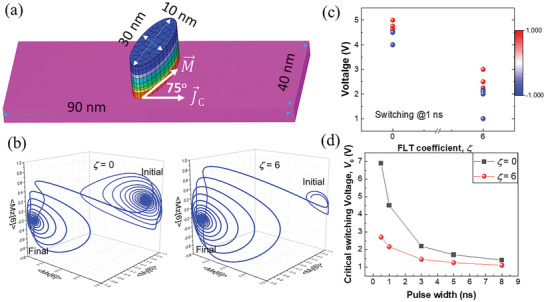
a) Structure of the 75°‐canted magnetic tunnel junction used in the simulation. b) Magnetization trajectories during switching. c) Color‐mapped plot of the switching behavior at 1 ns, where red and blue dots indicate switched and un‐switched devices, respectively. d) Pulse width dependence of the critical switching voltage for canted spin–orbit torque devices with *ζ* = 0 and *ζ* = 6.

To demonstrate the significant role of the high *ζ* in the triple‐domain‐structured (100) RuO_2_ in realizing high‐energy‐efficient SOT‐MRAM applications, we conducted a micromagnetic simulation of a canted SOT device. In this simulation, the in‐plane magnetic easy‐axis ($\vec{M}$) of the in‐plane magnetic tunnel junction is canted by 75° relative to the ${\vec{J}}_{C}$ (**Figure** **5**a). The stack structure and mesh were designed as described previously^[^ ^6^, ^13^ ^]^ (Section S9, Supporting Information). Herein, the channel layer in the SOT device is modeled as triple‐domain structured (100) RuO_2_, with a small spin Hall angle (α_H_) of 0.01 and high *ζ* of 6. We compared the simulation results with those for a device exhibiting negligible FL torque (*ζ* = 0). Figure 5b shows the magnetization trajectories under an external 8 ns‐pulse voltage of 1.5 V for devices with *ζ* = 0 and *ζ* = 6. For the device with *ζ* = 6, the magnetization polarity changed immediately after the torque was exerted in the initial state, quickly switching to the final state. In contrast, the device with *ζ* = 0 exhibited multiple precessions before and after the magnetization reversal. This leads to a faster switching time for the device with *ζ* = 6. Figure 5c shows a color‐mapped plot of the switching behavior at 1 ns, where red and blue dots represent switched and unswitched devices, respectively, for canted SOT devices with *ζ* = 0 and *ζ* = 6. The critical switching voltage (*V*_c_), evaluated at the point where the dot changes color, is 2.16 V for the device with *ζ* = 6, while *V*_c_ = 4.52 V for the device with *ζ* = 0. The error in *V*_c_ can be considered as the voltage step (0.1 V surrounding the switching region, where the dot changes color from blue to red. This error is very small compared with that of *V*_c_, and the voltage reduction because of the change in *ζ* (e.g., 4.52/2.16 times at a 1‐ns pulse) ensures the accuracy of the simulation results. Figure 5d shows the pulse‐width dependence of *V*_c_ for canted SOT devices with *ζ* = 0 and *ζ* = 6. For all pulse widths under simulation, the device with *ζ* = 6 requires a smaller *V*_c_ than the device with *ζ* = 0. As the pulse width decreases, the *V*_c_ required for stable bipolar switching is considerably lower, with a reduction factor of 2.56 at a 0.5 ns pulse for the device with *ζ* = 6. This demonstrates that utilizing triple‐domain‐structured (100) RuO_2_ with *ζ* = 6 significantly enhances the energy‐efficiency of SOT‐MRAM applications.

In the last section, we discussed the possible origins of the SOT, which can arise from 1) conventional SHE, 2) interfacial Rashba effect, and 3) SSE. To exclude the contribution of the interfacial Rashba effect, we prepared a control sample: RuO_2_ (4)/Ru(0.5)/Co_20_Fe_60_B_20_ (1.2)/MgO (1.3)/Ta (1.0) (hereafter RuO_2_/Ru/CFB), in which the interfacial conditions were modified. The *R*_ω_ and *R*_2ω_ curves for both RuO_2_/CFB‐ and RuO_2_/Ru/CFB‐based devices showed no significant changes, implying that the interfacial Rashba effect is negligible (Section S10, Supporting Information). Thus, the origins of the SOT were attributed to the SHE and SSE, with the total SHC expressed as: $\sigma^{\mathrm{tot}}=\sigma^{\mathrm{SHE}}+\sigma^{\mathrm{SSE}}∼J_{S\_Z}^{\mathrm{SHE}}+J_{S\_Z}^{\mathrm{SSE}}$, where σ^tot^ is the total SHC, and σ^SHE^ and σ^SSE^ represent the SHC induced by the SHE and SSE, respectively. The magnitude of these contributions is proportional to the spin current generated in the *Z*‐direction under the SHE ($J_{S\_Z}^{\mathrm{SHE}}$) and SSE ($J_{S\_Z}^{\mathrm{SSE}}$). Since the SHE is T_even and the SSE is T_odd, the components in the SHC tensors differ accordingly. Thus, the total SHC can be expressed as:

(12) $$\sigma^{\mathrm{tot}}∼\frac{3}{2}\left(c-a\right)J_{C}+\frac{3}{2}\left(C-A\right)J_{C}$$

where *A, C, a*, and *c* are the components of the SHC tensors corresponding to the SHE (first term) and SSE (second term), as reported in a previous study^[^ ^19^ ^]^ (Section S3, Supporting Information).

In principle, these parameters (*a, c, A*, and *C*) can be determined through density functional theory calculations with an approximation of the scattering rate (Γ), as reported in previous studies.^[^ ^15^, ^19^, ^24^ ^]^ In our RuO_2_ (100) system, the conductivity was ≈4000 Ω^−1^ cm^−1^, which is close to the value corresponding to a Γ of ≈50 meV reported in.^[^ ^19^ ^]^ Therefore, we estimated the contributions of the SHE (first term in Equation 12) and SSE (second term in Equation 12) by referring to the calculated values for the T_even SHE (*a* = −0.3777, *c* = 0.1277) and T_odd SSE (*A* = 0.4252, *C* = 1.9156) in ref. [19] The results indicate that the SSE accounted for 79% of the total spin generation, whereas the SHE contributed 21%, confirming that the SSE was the primary mechanism responsible for the observed SOTs in this system. In addition, other effects, such as spin scattering^[^ ^32^ ^]^ and/or spin swapping,^[^ ^33^ ^]^ may also affect the SOT. The spin‐scattering effect, which arises from the scattering of spin currents at the interfaces between the FM and adjacent oxide layers, has been reported to play a significant role in perpendicularly magnetized systems with ultrathin FM layers, where strong spin‐scattering can substantially impact the SOT.^[^ ^32^ ^]^ However, in the in‐plane magnetization system investigated in this study, this effect is expected to be relatively minor. For the spin‐swapping effect, weak disorder and a long carrier mean free path relative to the FM layer thickness are required,^[^ ^33^ ^]^ which are unlikely conditions in this system.^[^ ^34^ ^]^ Nevertheless, further investigations are required to fully elucidate these potential contributions.

## 4 Conclusion

In summary, we thoroughly investigated the SSE in collinear antiferromagnetic RuO_2_ (100) with a well‐controlled triple‐domain structure, revealing several key findings. The SHC was determined to be independent of the $\vec{N}$ because of the triple‐domain structure, leading to equal spin‐current generation when the *J_C_* was applied along the ${\left[1\bar{1}00\right]}_{\mathrm{Sub}}$ and ${\left[11\bar{2}0\right]}_{\mathrm{Sub}}$ directions. This enabled a precise evaluation of both the SL and FL torques in the RuO_2_/CFB bilayer, where RuO_2_ served as a spin‐current source, generating both *H*_SL_ and *H*_FL_ fields that acted on the magnetization in the Co_20_Fe_60_B_20_ film. The estimated SOT efficiencies, ξ_SL_ = −0.011 ± 0.001 and ξ_*FL*_ = −0.066 ± 0.001 at *J*_C_ = 6.87 × 10^10^ A m^−2^, clearly demonstrated that the FL torque was the dominant component, being six times stronger than the SL torque. The significant role of a high *β* (= 6) enabled a 2.56‐fold reduction in the critical switching voltage at 0.5 ns pulse in the canted SOT device, as demonstrated by micromagnetic simulations. The origins of the observed SOT were attributed to both the SSE and SHE, with the SSE contributing up to 79% of the total SHC in this system. Our experimental findings, leveraging the SSE and supported by symmetry analysis of the SHC, will contribute to the advancement of antiferromagnetic spintronics and development of high‐energy‐efficient SOT‐MRAM applications.

## Conflict of Interest

The authors declare no conflict of interest.

## Author Contributions

T.V.A.N. performed conceptualization, and methodology and wrote the original draft. Investigation performed by T.V.A.N., H.N., V.T.N.H., S.D.G., Y.S., D.V., and Y.E. Funding acquisition by T.V.A.N., Y.S., H.N., S.I., and T.E. Supervision by T.E. Writing—review and editing by all authors.

## Supporting information



Supporting Information

## Data Availability

The data that support the findings of this study are available on request from the corresponding author. The data are not publicly available due to privacy or ethical restrictions.
