# Supplementary material for: Enhanced Field‐Like Torque Generated from the Anisotropic Spin‐Split Effect in Triple‐Domain RuO2 for Energy‐Efficient Spin–Orbit Torque Magnetic Random‐Access Memory
Source: Adv Sci (Weinh). 2025 Feb 28;12(16):2413165. doi: 10.1002/advs.202413165 (PMC12407376; doi:10.1002/advs.202413165)
Supplement: Supplementary file 1 — Supporting Information [file ADVS-12-2413165-s001.docx]

**Supplementary information**

**Enhanced field-like torque generated from the anisotropic spin-split effect in triple-domain RuO_2_ for energy-efficient spin–orbit torque magnetic random-access memory**

**T. V. A. Nguyen,^1,2^ H. Naganuma,^1-4^ T. N. H. Vu,^5^ S. DuttaGupta,^1,6,7^ Y. Saito,^2^ D. Vu,^8^
Y. Endo,^1,9^ S. Ikeda,^1,2^ T. Endoh^1,2,7,9^**

^1^Center for Science and Innovation in Spintronics (Core Research Cluster), Tohoku University, Sendai, JAPAN

^2^Center for Innovative Integrated Electronic Systems, Tohoku University, Sendai, JAPAN

^3^Institute for Advanced Study, Nagoya University, Nagoya, JAPAN

^4^ Institute of Materials and Systems for Sustainability, Nagoya University, Nagoya, JAPAN

^5^Institute for Materials Research, Tohoku University, Sendai, JAPAN

^6^Saha Institute of Nuclear Physics, West Bengal, INDIA

^7^Research Institute of Electrical Communication, Tohoku University, Sendai, JAPAN

^8^Institute of Physics, Vietnam Academy of Science and Technology, Hanoi, VIETNAM ^9^Graduate School of Engineering, Tohoku University, Sendai, JAPAN

E-mail: nguyen.thi.van.anh.e7@tohoku.ac.jp

**S1: Sample Fabrication:**

RuO_2_ (100) was prepared on an *α*-Al_2_O_3_ (0001) substrate at 300^o^C by reactive sputtering where the percentage of oxygen gas flow (*P*_Oxygen_) was 30% in a mixture of Argon and Oxygen gases. The base pressure was 3 🞨 10^-6^ Pa and the reactive pressure was 0.13 Pa with a power of 150 W. Then RuO_2_ (4 nm)/Co_20_Fe_60_B_20_ (1.2 nm)/MgO (1.3 nm)/Ta (1.0 nm) stack films were prepared on *α*-Al_2_O_3_ (0001) by DC and RF sputtering. The numbers in parentheses represent the nominal thickness of the nanometer (Fig. S1).

Subsequently, the stack films were patterned into Hall-bar devices by photolithography and Ar-ion milling. Electrical contacts of Cr(10)/Au(100) were made by photolithography, sputtering, and lift-off. The size of the Hall bar in the device at *β* = 0° ($\vec{J}_{C}\text{ }\text{}\text{}\left. [11\bar{2}0 \right]_{\mathrm{Sub}}$) is 8.3 μm × 49.3 μm and that in the device at *β* = 90° ($\vec{J}_{C}\text{ }\text{ }\text{}\text{}\left. [1\bar{1}00 \right]_{\mathrm{Sub}}$) is 10.0 μm × 50.0 μm.


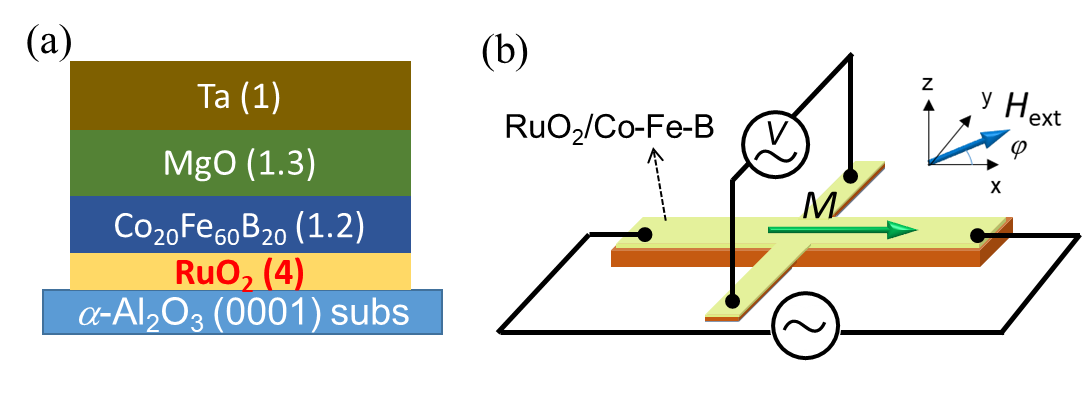


Fig. S1. (a) The stacking structure of a RuO_2_/Co-Fe-B/MgO/Ta film and (b) schematic image of a Hall device for harmonic Hall measurement under a rotated magnetization field *H*_ext_.

**S2: XAS spectra of RuO_2_:**

To confirm the oxidation state of the RuO_2_ layer, surface-sensitive soft X-ray absorption spectra (XAS) of Ru *M*_3_ and *M*_2_ and XAS at the oxygen *K*-edge were collected. Fig. S2 (a) shows the XAS of Ru *M*_3_ and *M*_2_ for the Ru film (in blue) and the RuO_2_ film (in red). The shoulders in the red spectrum could be attributed to the Ru^4+^ state in RuO_2_ [1, 2]. Fig. S2 (b) shows the XAS at the Oxygen *K*-edge for the RuO_2_ film (in red). Two peaks A and B could be attributed to the excitation of the O 1*s* core electrons into hybridized states between O 2*p* and Ru 4*d* *t*_2g_ and *e*_g_ states, respectively [3]. These results confirmed the formation of the RuO_2_ film on the *α*-Al_2_O_3_ (0001) substrate.


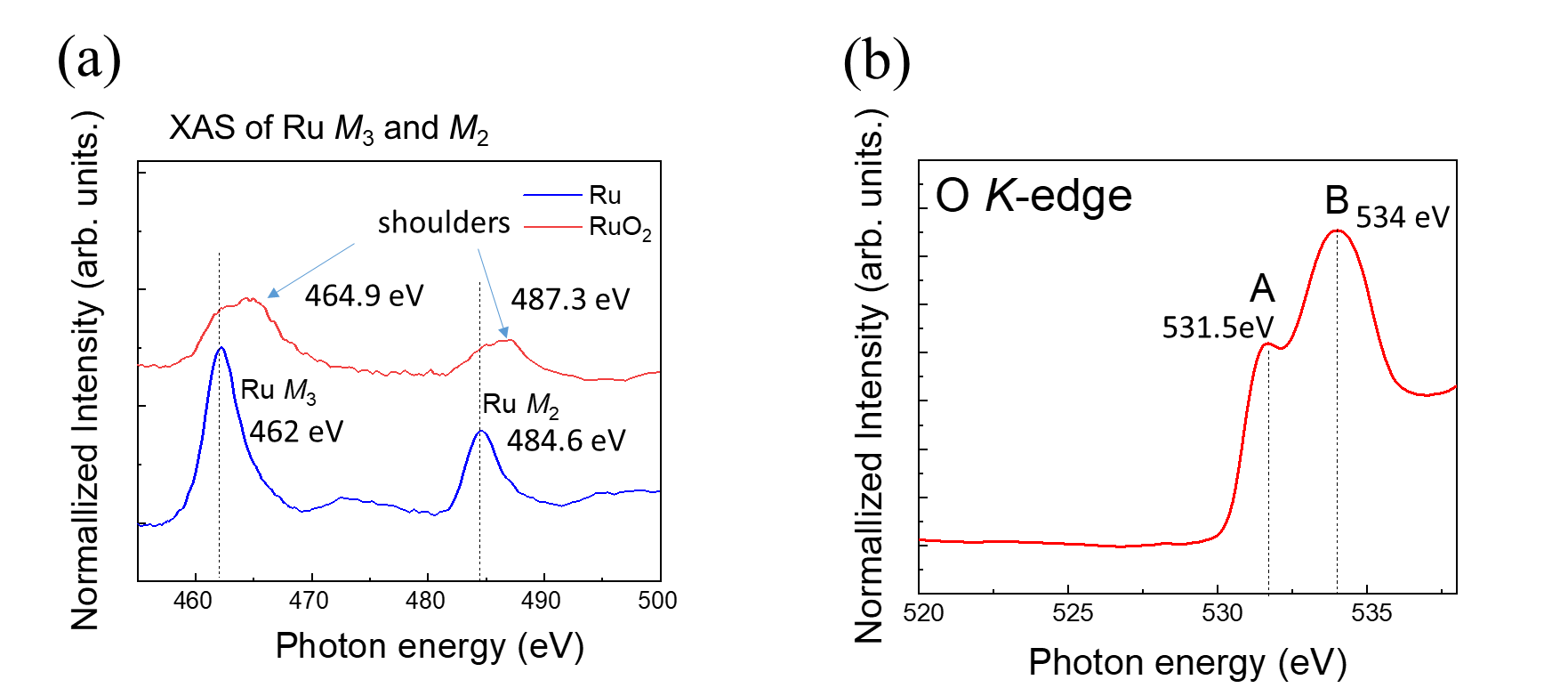


Fig. S2. (a) XAS of Ru M_3_ and M_2_ for the Ru film (in blue) and the RuO_2_ film (in red). (b) XAS at the oxygen *K*-edge for RuO_2_ film (red).

**S3: Calculation of Spin Hall conductivity (SHC) tensor:**

In principle, SHC can be estimated from the electronic structure calculations in combination with generating a tight-binding Hamiltonian for the system using first-principles calculations. More clearly, the time-reversal odd and even parts of SHC are given by Kubo's formula [4, 5]:

$\sigma_{ij}^{k}=-\frac{e\hbar}{\pi}\int\frac{d^{3}\vec{k}}{{(2\pi)}^{3}}\sum_{n,m} \frac{\Gamma^{2}\mathrm{Re}\left( \left\langle n\vec{k} | j_{i}^{k} | m\vec{k} \right\rangle\left\langle m\vec{k} | v_{j} | n\vec{k} \right\rangle\right)}{\left[ \left( E_{F}-E_{n\vec{k}} \right)^{2}+\Gamma^{2} \right]\left[ \left( E_{F}-E_{m\vec{k}} \right)^{2}+\Gamma^{2} \right]}$,

$\sigma_{ij}^{k}=-\frac{2e}{\hbar}\int\frac{d^{3}\vec{k}}{{(2\pi)}^{3}}\sum_{n^{'}\neq n} \frac{\mathrm{Im}\left( \left\langle n\vec{k} | j_{i}^{k} | n^{'}\vec{k} \right\rangle\left\langle n^{'}\vec{k} | v_{j} | n\vec{k} \right\rangle\right)}{\left( E_{n\vec{k}}-E_{n^{'}\vec{k}} \right)^{2}}$,

where $j_{i}^{k}$ is the spin-current operator, $f_{n\vec{k}}$ is the Fermi-Dirac distribution for band *n* and wave vector $\vec{k}$, and Γ is the scattering rate.

In this study, the crystal structure of RuO_2_ (100) has the same symmetry as that of RuO_2_ (001) in the paper [6,7]. Therefore, RuO_2_ (100) has a Néel vector parallel with [001] axis, then the spin Hall conductivity tensor has a form [6,7]:

|  | $\boldsymbol{\sigma}^{\boldsymbol{x}}$ | $\boldsymbol{\sigma}^{\boldsymbol{y}}$ | $\boldsymbol{\sigma}^{\boldsymbol{z}}$ |
| --- | --- | --- | --- |
|  | $\left( \begin{matrix} \sigma_{xx}^{x} & \sigma_{xy}^{x} & \sigma_{xz}^{x} \\ \sigma_{yx}^{x} & \sigma_{yy}^{x} & \sigma_{yz}^{x} \\ \sigma_{zx}^{x} & \sigma_{zy}^{x} & \sigma_{zz}^{x} \end{matrix} \right)$ | $\left( \begin{matrix} \sigma_{xx}^{y} & \sigma_{xy}^{\boldsymbol{y}} & \sigma_{xz}^{\boldsymbol{y}} \\ \sigma_{yx}^{\boldsymbol{y}} & \sigma_{yy}^{\boldsymbol{y}} & \sigma_{yz}^{\boldsymbol{y}} \\ \sigma_{zx}^{\boldsymbol{y}} & \sigma_{zy}^{y} & \sigma_{zz}^{\boldsymbol{y}} \end{matrix} \right)$ | $\left( \begin{matrix} \sigma_{xx}^{\boldsymbol{z}} & \sigma_{xy}^{\boldsymbol{z}} & \sigma_{xz}^{\boldsymbol{z}} \\ \sigma_{yx}^{\boldsymbol{z}} & \sigma_{yy}^{\boldsymbol{z}} & \sigma_{yz}^{\boldsymbol{z}} \\ \sigma_{zx}^{\boldsymbol{z}} & \sigma_{zy}^{\boldsymbol{z}} & \sigma_{zz}^{\boldsymbol{z}} \end{matrix} \right)$ |
| RuO_2_(100)  X[010]  Y[001] | $\left( \begin{matrix} \boldsymbol{0} & \boldsymbol{0} & \boldsymbol{0} \\ \boldsymbol{0} & \boldsymbol{0} & \boldsymbol{B} \\ \boldsymbol{0} & \boldsymbol{A} & \boldsymbol{0} \end{matrix} \right)$ | $\left( \begin{matrix} \boldsymbol{0} & \boldsymbol{0} & \boldsymbol{C} \\ \boldsymbol{0} & \boldsymbol{0} & \boldsymbol{0} \\ \boldsymbol{C} & \boldsymbol{0} & \boldsymbol{0} \end{matrix} \right)$ | $\left( \begin{matrix} \boldsymbol{0} & \boldsymbol{A} & \boldsymbol{0} \\ \boldsymbol{B} & \boldsymbol{0} & \boldsymbol{0} \\ \boldsymbol{0} & \boldsymbol{0} & \boldsymbol{0} \end{matrix} \right)$ |

where *A*, *B*, and *C* are components of SHC tensors. Their values are exchanged with those in the tensor for RuO_2_ (001) in [3] due to the rotation tensor while rotating the coordinate for differently oriented RuO_2_ film.

The spin Hall conductivity of Domain 1 with [001] axis tilts an angle $\theta$ with *Y* axis in a Cartesian coordinate system is obtained as follows:

$$\sigma_{(\theta)ij}^{s,k}\boldsymbol{=}\sum_{\boldsymbol{lmn}} \boldsymbol{D}_{\boldsymbol{il}}\boldsymbol{D}_{\boldsymbol{jm}}\boldsymbol{D}_{\boldsymbol{kn}}\sigma_{(0)lm}^{s,n}$$

where ***D*** is the transformation matrix, $\boldsymbol{D}=\left( \begin{matrix} \mathbf{cos}\boldsymbol{\theta} & \boldsymbol{-}\mathbf{sin}\boldsymbol{\theta} & \boldsymbol{0} \\ \mathbf{sin}\boldsymbol{\theta} & \mathbf{cos}\boldsymbol{\theta} & \boldsymbol{0} \\ \boldsymbol{0} & \boldsymbol{0} & \boldsymbol{1} \end{matrix} \right)$. Thus,

$$\boldsymbol{\sigma}^{\boldsymbol{x}}=\left( \begin{matrix} \boldsymbol{0} & \boldsymbol{0} & \boldsymbol{0} \\ \boldsymbol{0} & \boldsymbol{0} & \boldsymbol{-C}\mathbf{sin}^{\boldsymbol{2}} \boldsymbol{\theta+B}\mathbf{cos}^{\boldsymbol{2}} \boldsymbol{\theta} \\ \boldsymbol{0} & \boldsymbol{-C}\mathbf{sin}^{\boldsymbol{2}} \boldsymbol{\theta+A}\mathbf{cos}^{\boldsymbol{2}} \boldsymbol{\theta} & \boldsymbol{0} \end{matrix} \right)$$

$$\boldsymbol{\sigma}^{\boldsymbol{y}}=\left( \begin{matrix} \boldsymbol{0} & \boldsymbol{0} & \boldsymbol{-B}\mathbf{sin}^{\boldsymbol{2}} \boldsymbol{\theta+C}\mathbf{cos}^{\boldsymbol{2}} \boldsymbol{\theta} \\ \boldsymbol{0} & \boldsymbol{0} & \boldsymbol{0} \\ \boldsymbol{-A}\mathbf{sin}^{\boldsymbol{2}} \boldsymbol{\theta+C}\mathbf{cos}^{\boldsymbol{2}} \boldsymbol{\theta} & \boldsymbol{0} & \boldsymbol{0} \end{matrix} \right)$$

$$\boldsymbol{\sigma}^{\boldsymbol{z}}=\left( \begin{matrix} \boldsymbol{0} & \boldsymbol{-B}\mathbf{sin}^{\boldsymbol{2}} \boldsymbol{\theta+A}\mathbf{cos}^{\boldsymbol{2}} \boldsymbol{\theta} & \boldsymbol{0} \\ \boldsymbol{-A}\mathbf{sin}^{\boldsymbol{2}} \boldsymbol{\theta+B}\mathbf{cos}^{\boldsymbol{2}} \boldsymbol{\theta} & \boldsymbol{0} & \boldsymbol{0} \\ \boldsymbol{0} & \boldsymbol{0} & \boldsymbol{0} \end{matrix} \right)$$

In the case of a single domain structure in RuO_2_ (100), SHC tensors are dependent on the angle $\theta$, shown in the above SHC tensors. However, in the case of RuO_2_ (100) with the triple-domain structure, the SHC becomes independent of the angle $\theta$ [Eq. (1) in the main text].

$$\sigma_{zy}^{x}=\sigma_{zy}^{x,Domain 1}+\sigma_{zy}^{x,Domain 2}+\sigma_{zy}^{x,Domain 3}$$

$\sigma_{zy}^{x}=-C{[sin}^{2} \theta+\sin^{2} \left( \theta+120 \right)+\sin^{2} \left( \theta+240 \right)]+A{[cos}^{2} \theta+\cos^{2} \left( \theta+120 \right)+\cos^{2} \left( \theta+240 \right)]=\frac{3}{2}(A-C)$ (S3_1)

The spin current is generated when the charge current is applied to the device. We define $\beta$ as the angle between the applied current $\vec{J}_{C}$ and *X-*direction (Fig. S3). Then, the spin currents along the *Z*-direction, which were experimentally measured in this study, can be expressed as:

$J_{S\_Z}^{\mathrm{tot}} = J_{S\_Z}^{X}+J_{S\_Z}^{Y} \sim{(\sigma_{zy}^{x}\sin\beta+\sigma}_{zx}^{y}\cos\beta)J_{C} = \frac{3}{2}\left( A-C \right)\sin\beta J_{C}+\frac{3}{2}\left( C-A \right)\cos\beta J_{C}$ (S3_2)

where the finite SHC components contributed to this spin current are listed in the table:

| SHC | Out-of-plane spin currents (along the *Z*-direction) |
| --- | --- |
| $\sigma_{zy}^{x}$ | $\frac{3}{2}\left( A-C \right)\sin\beta$ |
| $\sigma_{zx}^{y}$ | $\frac{3}{2}\left( C-A \right)\cos\beta$ |

Now, we show the SHC components for three devices in this study. The SHC component for the device with $\beta={90}^{o}$, i.e., $\vec{J}_{C}\text{ }\text{}\text{}\text{ }\vec{N}_{1}$ ($\vec{J}_{C}\text{ }\text{}\text{}\left. [1\bar{1}00 \right]_{\mathrm{Sub}}$) is:

| SHC | SHC for the device with $\beta={90}^{o}$ ($\vec{J}_{C}\text{ }\text{}\text{}\text{ }\vec{N}_{1}$) | |
| --- | --- | --- |
| $\sigma_{zy}^{x}$ | $\frac{3}{2}\left( A-C \right)$ | Spin current *X*-polarized in *Z*-direction |

The SHC component for the device with $\beta=0^{o}$, i.e..$\vec{J}_{C}\text{ }\text{}\text{ }\vec{N}_{1}$ ($\vec{J}_{C}\text{ }\text{}\text{}\left. [11\bar{2}0 \right]_{\mathrm{Sub}}$) is:

| SHC | SHC for the device with $\beta=0^{o}$ ($\vec{J}_{C}\text{ }\text{}\text{ }\vec{N}_{1}$) | |
| --- | --- | --- |
| $\sigma_{zx}^{y}$ | $\frac{3}{2}\left( C-A \right)$ | Spin current *Y*-polarized in *Z-*direction |

The SHC components for the device with $\beta={45}^{o}$ are:

| SHC | SHC for the device with $\beta={45}^{o}$ | |
| --- | --- | --- |
| $\sigma_{zy}^{x}$ | $\frac{3}{2}\left( A-C \right)\frac{\sqrt{2}}{2}$ | Spin current *X*-polarized in *Z-*direction |
| $\sigma_{zx}^{y}$ | $\frac{3}{2}\left( C-A \right)\frac{\sqrt{2}}{2}$ | Spin current *Y*-polarized in *Z*-direction |

These components belong to the total spin current in the *Z*-direction generated by the charge current applied in ${45}^{o}$ from $\left. [11\bar{2}0 \right]_{\mathrm{Sub}}$ direction with the orthogonal polarization. As a result, the SHC and the spin current for the three devices are identical as shown in the main text.

In the last part of the manuscript, we discussed the possible origins of the SOT in the triple-domain structure RuO_2_ (100) with the contribution of the SSE and SHE based on the values of the *A*, *B*, and *C* components of SHE tensors. The *A*, *B*, and *C* are components of SHC tensors that could be evaluated by solving Kubo’s formula with the input of the scattering rate (Γ), which can be estimated by comparing it with the experimental conductivity [7]. In this study, the RuO_2_ (100) film has a conductivity of ~4000 Ω^-1^cm^-1^, which is close to the value reported in ref. [7] with the scattering rate (Γ) of 50 meV. As a result, the electronic structure and parameters to determine SHC in Kubo's formula would be close to those in ref. [7]. The further calculation would not significantly change SHC results and, therefore, we could refer to ref. [7] for the last part of the manuscript.


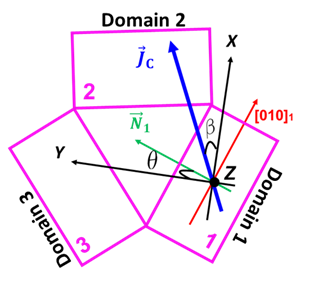


**Fig. S3. The current applied to the sample at a certain angle *β* versus the *X* axis, which is also shown in Fig. 2 (b) in the main text.**

**S4: Magnetic property and anomalous Hall resistance of RuO_2_/Co-Fe-B:**

The static magnetic properties of the samples were investigated by a vibrating sample magnetometer (VSM) under an out-of-plane field (*H*_OP_) and in-plane magnetic field (*H*_IP_) (Fig. S4 (a)). The results show that the stack film has an in-plane easy axis. The evaluated saturation magnetization is *M*_S_ = 1500.4 emu/cc.

For the Hall bar devices, the anomalous Hall resistance curve in the out-of-plane field was measured (Fig. S4 (b) and (c)). From the linear fit to the Hall resistance curve at the low field and high field regions, the anomalous Hall resistance (*R*_AHE_ = 7.36 Ω), and the effective anisotropy field (*μ*_0_$H_{K}^{\mathrm{eff}}$ = -269.12 mT) were evaluated for the device with *β* = 90° ($\vec{J}_{C}\text{ }\text{}\text{}\left. [1\bar{1}00 \right]_{\mathrm{Sub}}$); and those for the device with *β* = 0° ($\vec{J}_{C}\text{ }\text{}\text{}\left. [11\bar{2}0 \right]_{\mathrm{Sub}}$) are *R*_AHE_ = 7.68 Ω, *μ*_0_$H_{K}^{\mathrm{eff}}$ = -560.6 mT. The change of $H_{K}^{\mathrm{eff}}$ with the *β* angle would suggest a change in the magnetic elastic anisotropy because a small change in the device fabrication-induced strain could result in a change in the bulk anisotropy [8], while other contributions from the interfacial anisotropy and demagnetization field would be negligible because we used the same stacking structure for all micrometer-sized devices. This speculation again calls for further investigation to give a clear answer to this. Since the value of $H_{K}^{\mathrm{eff}}$ directly relates to the evaluation of *H*_SL_ following Eq. (7) in the main text, we need to evaluate the exact value of $H_{K}^{\mathrm{eff}}$for each device to give a correct value of *H*_SL_.


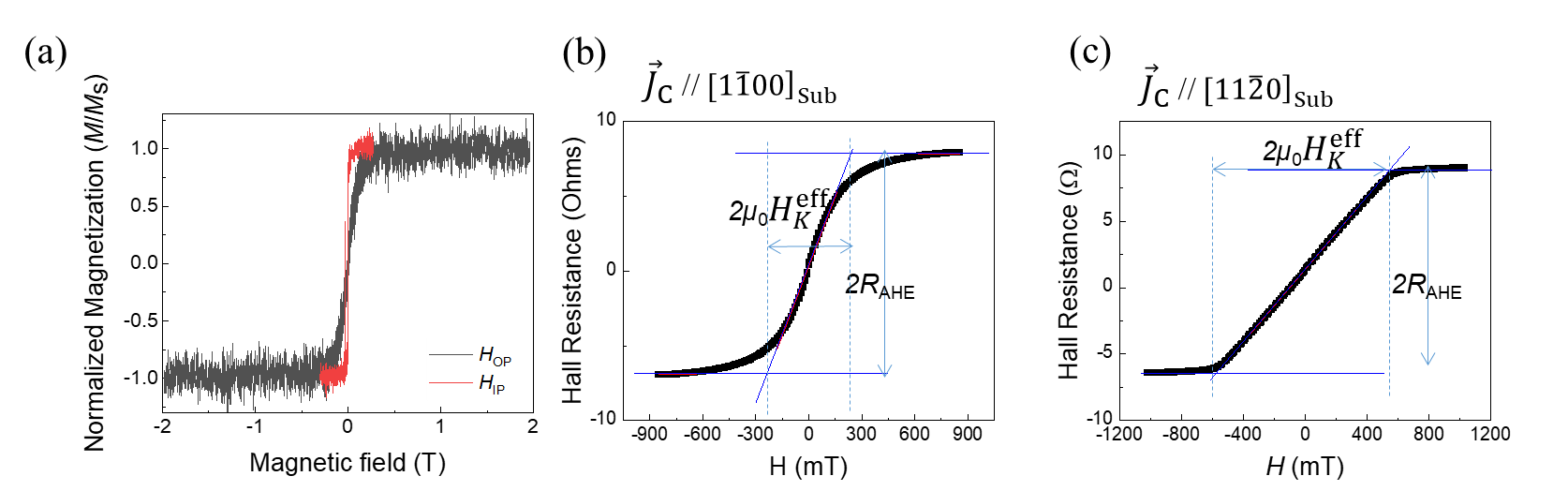


Fig. S4. (a) Magnetization curves of the stack film. The anomalous Hall resistance for (b) the device with ${\vec{\boldsymbol{J}}}_{\mathbf{C}}\text{}\text{}\left. \mathbf{[1}\bar{\mathbf{1}}\mathbf{00} \right]_{\mathbf{Sub}}$and that for (c) the device with ${\vec{\boldsymbol{J}}}_{\mathbf{C}}\text{ }\text{}\text{}\left. \mathbf{[11}\bar{\mathbf{2}}\mathbf{0} \right]_{\mathbf{Sub}}$.

**S5: Resistivity of RuO_2_ and Co-Fe-B:**

The resistivity (*ρ*) of each layer is determined by measuring sheet resistance on a series of blanket films. Fig. S5 shows the inverse of the measured sheet resistance as a function of RuO_2_ layer thickness (*t*_RuO2_) and Co-Fe-B layer thickness (*t*_CFB_). By fitting with a linear function [9], the resistivity of RuO_2_ and Co-Fe-B layers is 247.4 µΩcm, and 162.7 µΩcm, respectively.


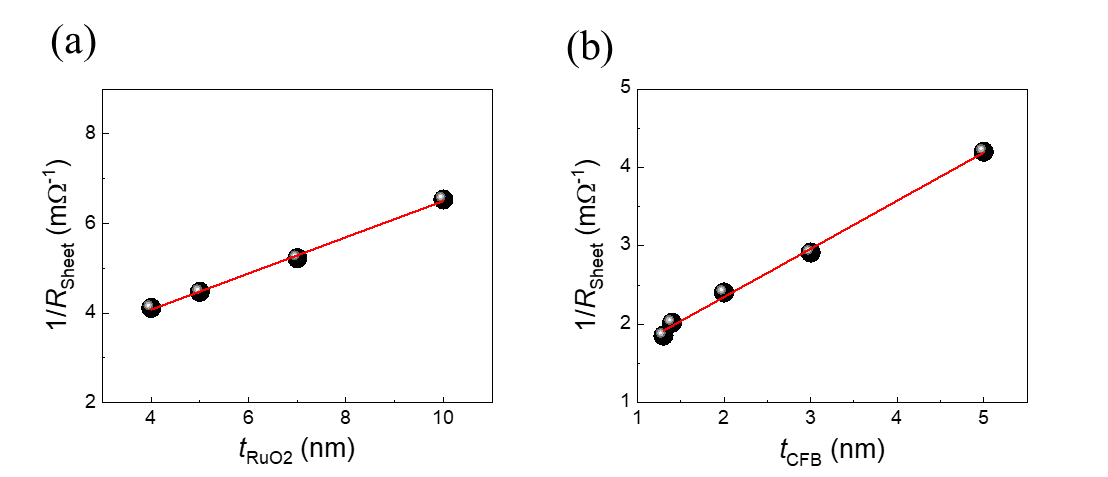


Fig. S5. (a) Inverse of Sheet resistivity vs. RuO_2_ layer thickness and (b) Co-Fe-B layer thickness.

**S6: Contribution of the Oersted field (*H*_OE_):**

*R*_2ω_ signal includes contributions from the Oersted field (*H*_OE_) related to the current applied to the device and the SOT effective fields (*H*_SL_ and *H*_FL_). *H*_FL_ could be calculated using the 2^nd^ term of Eq. (7), after subtracting the contribution of *H*_OE_ calculated using: *H*_OE_=*I*/2*w*, where *I* is the current flowing into the RuO_2_ layer, and *w* is the channel width [10, 11].

Figure S6 shows the current density (*J*_C_) dependence of *H*_FL_ (in red), and *H*_OE_ (in black) for the devices with (a) $\vec{J}_{C}\text{ }\text{}\text{}\left. [1\bar{1}00 \right]_{\mathrm{Sub}}$ and (b) $\vec{J}_{C}\text{ }\text{}\text{}\left. [11\bar{2}0 \right]_{\mathrm{Sub}}$. The results show that *H*_OE_ and *H*_FL_ have the same direction, which is similar to the reported result for the (101) RuO_2_/Py system [7], and other devices using the same measurement set-up as we used [11 *and others therein*]. Herein, *H*_OE_ only contributed about ~21% to ~24% compared with *H*_FL_ which is consistent with the previous paper [11 and others therein]. In our measurement, we have subtracted the contribution of *H*_OE_ to obtain the actual value of *H*_FL_ for every device.

**
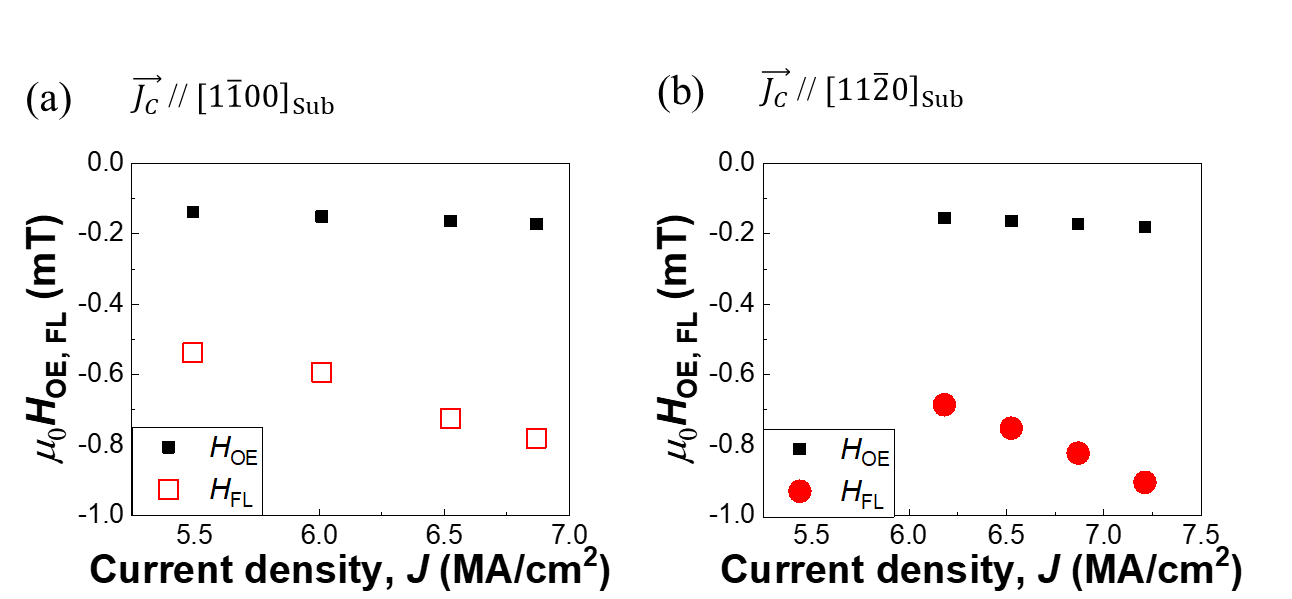
**

Fig. S6. Current density (*J*_C_) dependence of *H*_FL_ (in red), and *H*_OE_ (in black) for the devices with (a) ${\vec{\boldsymbol{J}}}_{\mathbf{C}}\text{ }\text{}\text{}\left. \mathbf{[1}\bar{\mathbf{1}}\mathbf{00} \right]_{\mathbf{Sub}}$ and (b) ${\vec{\boldsymbol{J}}}_{\mathbf{C}}\text{ }\text{}\text{}\left. \mathbf{[11}\bar{\mathbf{2}}\mathbf{0} \right]_{\mathbf{Sub}}$.

**S7: SOT in (110) RuO_2_/CFB devices:**

We prepared control devices based on the stacking film: **(110)-RuO_2_** (5)/ Co_20_Fe_60_B_20_ (2)/MgO(1.3)/Ta(1) stacked on a (110) TiO_2_ substrate, namely **DV_110RuO_2_**.

Figure S7 (a) shows the RHEED image for a 5 nm RuO_2_ grown on the (110) TiO_2_ substrate. The clear dots/streaks feature is observed, indicating an atomically flat surface morphology under a 3D growth mode [12] of the RuO_2_ (110) film grown on the TiO_2_ (110) substrate.

Figures S7 (b) and (c) show the first and second harmonic Hall resistance curves for the device DV_110RuO_2_. The 1^st^ harmonic Hall resistance could be measured while the 2^nd^ harmonic Hall resistance could not be detected, i.e., the SOT fields are negligible in this sample. This result is consistent with the theoretical paper [6], in which it was speculated that there is no SSE-induced SOT in the RuO_2_ (110)-based device because there is no out-of-plane spin current to be generated in RuO_2_ (110).


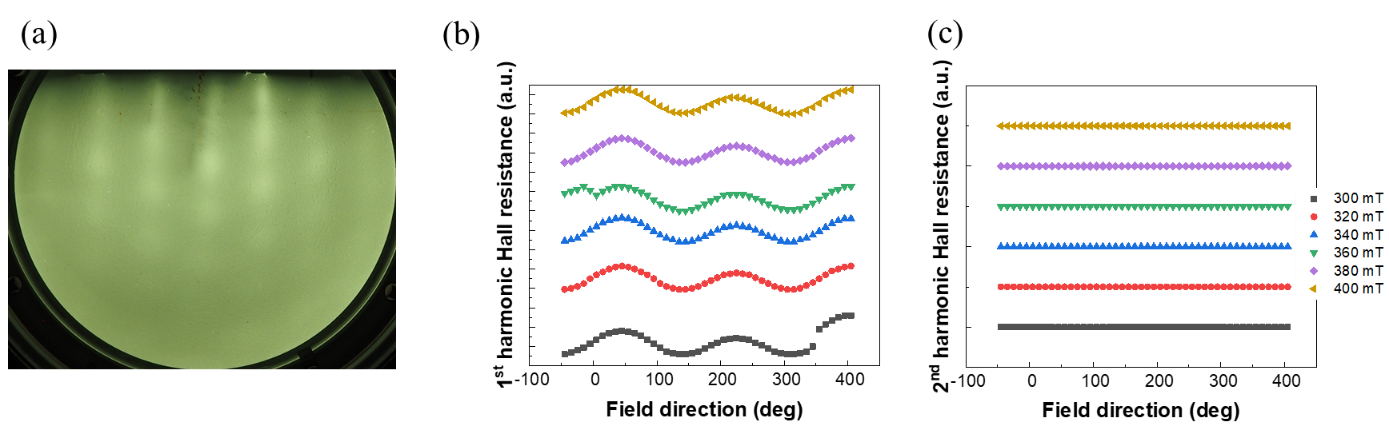


Fig. S7. (a) RHEED image of a (110) RuO_2_ on (110) TiO_2_ substrate. The first and second harmonic Hall resistance curves for the device DV_110RuO_2_ are shown in (a) and (b), respectively

**S8: SOT in the device with *β* = 45°:**

We showed the investigation of the angle dependence of the SSE-induced SOT in the triple-domain structured (100) RuO_2_/CFB bilayer.

From the SHC tensor calculation, the spin currents along *X-*, *Y-*, *Z-*axes are as follows (also shown in the main text):

$J_{S\_X}^{Z} \sim\sigma_{xy}^{z}J_{C}\sin\beta$*=* $\frac{3}{2}(A-B)\sin\beta J_{C}$ (2)

$J_{S\_Y}^{Z} \sim\sigma_{yx}^{z}J_{C}\cos\beta$ *=* $\frac{3}{2}(B-A)\cos\beta J_{C}$ (3)

$J_{S\_Z}^{\mathrm{tot}} = J_{S\_Z}^{X}+J_{S\_Z}^{Y}\sim{(\sigma_{zy}^{x}\sin\beta+\sigma}_{zx}^{y}\cos\beta)J_{C} = \frac{3}{2}\left( A-C \right)\sin\beta J_{C}+\frac{3}{2}\left( C-A \right)\cos\beta J_{C}$(4)

where the spin current in *X-* and *Y-*directions is polarized in the *Z* direction (Eqs. (2) and (3)), and the spin current in the *Z*-direction (out-of-plane spin current) in (Eq. (4)) is polarized in *X-* (the first term of Eq. (4)) and *Y-*directions (the second term of Eq. (4)), that is in-plane polarization.

The SOT is generated by the spin current in the *Z*-direction, $J_{S\_Z}^{\mathrm{tot}}$. For the device with $\beta={90}^{o}$, this spin current $J_{S\_Z}^{\mathrm{tot}} \sim\frac{3}{2}(A-C)J_{C}$ is polarized in the *X*-direction. For the device with $\beta=0^{o}$, this spin current $J_{S\_Z}^{\mathrm{tot}} \sim\frac{3}{2}(C-A)J_{C}$ is polarized in the *Y*-direction. However, it should be noted that for the device with a certain $\beta$ angle, the spin current in the *Z* direction includes two components that are polarized in both the *X-*direction (1^st^ term) and the *Y-*direction (2^nd^ term). In the Harmonic Hall measurement, we could detect the total SOT from the spin current with both components. The magnitude of this spin current can be calculated using the Pythagorean theorem because of their vector nature:

$$J_{S_{Z}}^{tot}=\sqrt{\left( \frac{3}{2}\left( A-C \right)\sin\beta J_{C} \right)^{2}+\left( \frac{3}{2}(C-A)\cos\beta J_{C} \right)^{2}}=\frac{3}{2}\left| (C-A) \right|J_{C}$$

This result implies that the SOT for a device with a certain *β* angle is also similar to those for *β* = 90°-device and *β* = 0°-device.

Experimentally, we fabricated the SOT in the *β* = 45°-device to evaluate the spin-orbit torques for comparison with those of *β* = 90°- device ($\vec{J}_{C}\text{ }\text{}\text{}\left. [1\bar{1}00 \right]_{\mathrm{Sub}}$ or $\vec{J}_{C}\text{ }\text{}\text{}\text{ }\vec{N}_{1}$) and *β* = 0°-device ($\vec{J}_{C}\text{ }\text{}\text{}\left. [11\bar{2}0 \right]_{\mathrm{Sub}}$, i.e., $\vec{J}_{C}\text{ }\text{}\text{ }\vec{N}_{1}$).

For the Hall bar device with *β* = 45°, the anomalous Hall resistance curve in the out-of-plane field was measured (Fig. S8 (a)), and the anomalous Hall resistance (*R*_AHE_ = 8.08 Ω), and the effective anisotropy field (*μ*_0_$H_{K}^{\mathrm{eff}}$ = -390.56 mT) were estimated. Figures S8 (b) and (c) show the first ($R_{\omega}$) and second ($R_{2\omega}$) harmonic Hall resistances for *β* = 45°-device. Figures S8 (d) and (e) show the linear fits for the first and the second terms of Eq. (7) in the main text, based on which we evaluated *H*_SL_ and *H*_FL_ for this device.


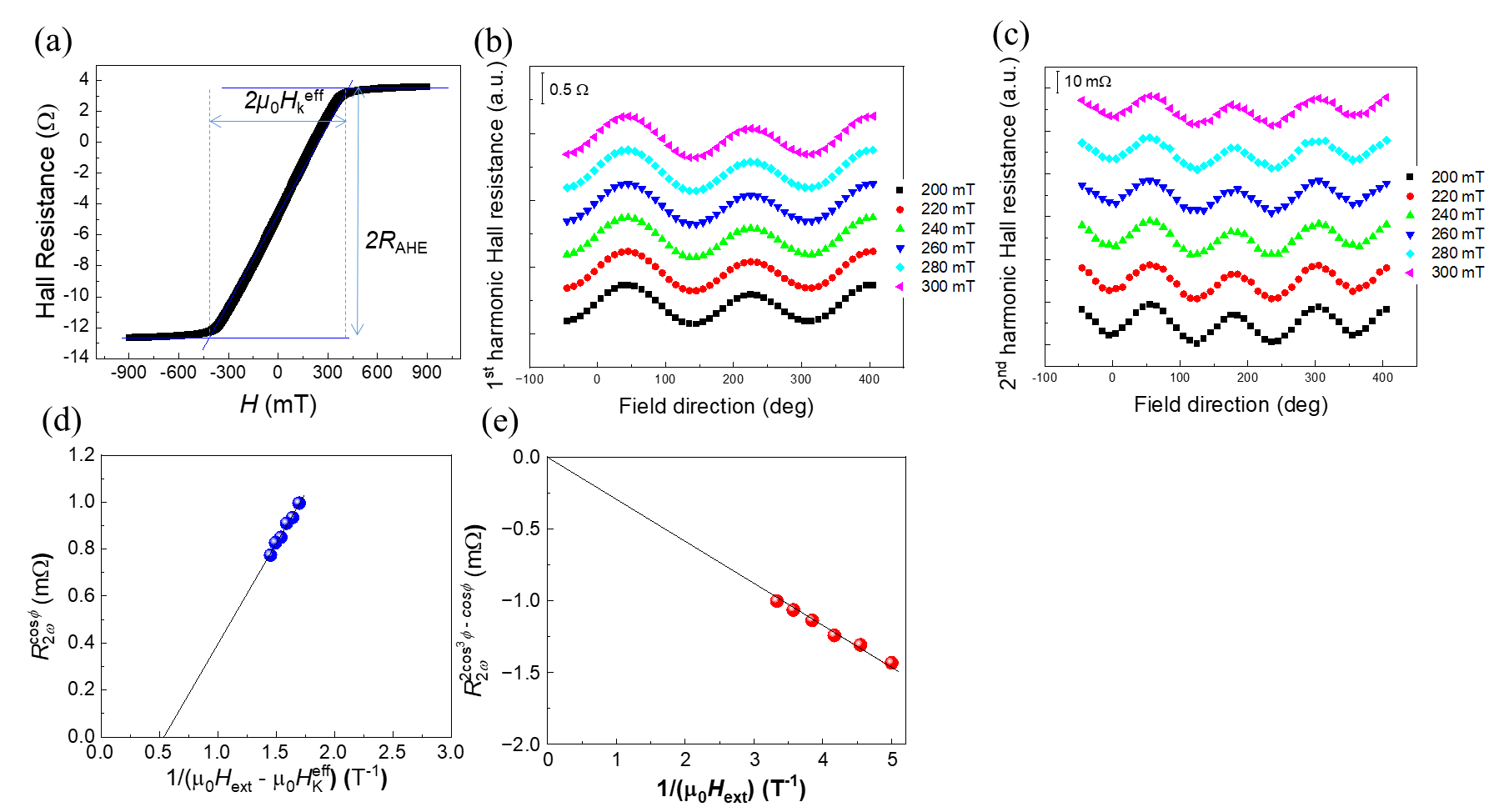


Fig. S8. (a) The anomalous Hall resistance for the device with *β* = 45°. The first and second harmonic Hall resistance curves for this device are shown in (b) and (c), respectively. The fits of first and second terms of eq. (7) are shown in (d) and (e).

*H*_SL_ and *H*_FL_ for *β* = 45°-device are listed in Table S1, in comparison with those for *β* = 90°- device and the *β* = 0°-device.

Table S1: *H*_SL_/*J* and *H*_FL_/*J* for *β* = 0°, 45°, 90°-devices

| Device | *H*_FL_/*J* (10^-3^T/(A/m^2^)) | *H*_SL_/*J* (10^-3^T/(A/m^2^)) |
| --- | --- | --- |
| *β* = 0° | -0.119±1.07% | -0.019±4.60% |
| *β* = 45° | -0.117±0.60% | -0.015±2.86% |
| *β* = 90° | -0.112±0.25% | -0.017±4.86% |

We evaluated SHC, i.e. the torque efficiency per unit electric field ($\xi_{SL,E}$ and $\xi_{FL,E}$), (also called spin torque conductivity) using equation [7]:

$$\xi_{SL(FL),E}=\frac{\hbar}{2e}\frac{\xi_{SL(FL)}}{\rho_{\mathrm{xx}}}$$

where ℏ is the reduced Plack constant, and *e* is the elementary charge, $\rho_{\mathrm{xx}}$ is the longitudinal resistivity of the RuO_2_ layer.

$\xi_{SL,E}$ and $\xi_{FL,E}$ for 4 nm thick triple-domain RuO_2_ (100) for the three devices are shown in Table S2:

Table S2: $\boldsymbol{\xi}_{\mathbf{SL,E}}$ and $\boldsymbol{\xi}_{\mathbf{FL,E}}$ for *β* = 0°, 45°, 90°-devices

| Device | $\xi_{FL,E}$  $\left( m \right)^{-1}$ | $\xi_{SL,E}$  $\left( m \right)^{-1}$ |
| --- | --- | --- |
| *β* = 0° | $\left( 2.7 0.1 \right){10}^{4}$ | $\left( 4.4 0.3 \right){10}^{3}$ |
| *β* = 45° | $\left( 2.5 0.1 \right){10}^{4}$ | $\left( 4.2 0.1 \right){10}^{3}$ |
| *β* = 90° | $\left( 2.8 0.1 \right){10}^{4}$ | $\left( 4.2 0.1 \right){10}^{3}$ |

The close experimental values between the three devices reproduce the suggestion from the theoretical calculation of SHC, proving the evidence for the SSE-induced SOT in the triple-domain structured (100) RuO_2_/Co-Fe-B bilayer. It should be noted that the experimentally evaluated values $\xi_{SL(FL),E}$ varied less than 10% with the possible changes in the interfacial spin transparency, and Spin Hall angle ($\theta_{\mathrm{SH}}$), and conductivity tensor, and so on between the three devices. Herein, we referred to the spin diffusion length in RuO_2_, ${}_{\mathrm{SHE}}$ = 2.6 nm from the previous report [7], which is less than the thickness under investigation in this study (4 nm RuO_2_), and therefore ${}_{SL(FL)}$ was used instead of $\theta_{\mathrm{SH}}$ following the drift-diffusion model: ${}_{SL(FL)}=\theta_{\mathrm{SH}}\left( 1-\mathrm{sech}\left( \frac{t_{RuO2}}{{}_{\mathrm{SHE}}} \right) \right)$ [13,14]. In addition, the longitudinal resistivity of the RuO_2_ layer $\rho_{\mathrm{xx}}$ was used because of a negligible transverse resistivity $\rho_{\mathrm{xy}}$ (due to the small *R*_AHE_ with a tiny change, and close geometrical factors of the Hall bar between three devices) following the conductivity tensor $\sigma_{\mathrm{xx}}=\rho_{\mathrm{xx}}/\left( {\rho_{\mathrm{xx}}}^{2}+{\rho_{\mathrm{xy}}}^{2} \right)$ [15,16]. In addition, the possible effects of defects, domain imperfections, and finite temperature may introduce variations between the experiment and theoretical results of SHC for each device. All these factors are the subjects of future study.

**S9: Micro-magnetic simulation for canted SOT devices:**

The stacking structure of the MTJ device is channel layer (5)/ free layer (1.48)/ MgO tunneling barrier (1.8)/ Co-Fe-B and Co-based synthetic ferrimagnetic reference layer (6) /top electrode (5). The numbers in parentheses represent the nominal thickness in nanometers. The SOT devices have a 30 × 10 nm^2^ elliptic MTJ placed on a 90 × 40 nm^2^ channel layer. Each layer was divided into a mesh of discretized cells with the size of 2 nm × 2 nm × *t* nm (*t* is the thickness of each layer). This size is smaller than the exchange length of the Co-Fe-B thin film to satisfy the calculation accuracy of the domain wall under the domain wall propagation model [17,18]. The easy axis of the MTJ is 75°-canted with the in-plane current ($\vec{J}_{C}$) flowing in the *x*-direction.

To simulate the effects of the field-like torque (FLT) on the magnetization switching, we employed the Landau-Lifshitz-Gilbert (LLG) equation (Eq. (S9_1)) that includes the precession torque (the first term), the damping torque (the second term) and spin torques with both of the Slonczewski-like (SL) torque (the third term) and FL torque (the fourth term) as shown below:

$\frac{\partial\vec{M}}{\partial t}=-\gamma\left[ \vec{M}\times\vec{H}_{\mathrm{eff}} \right]+\frac{\alpha}{M_{S}}\left[ \vec{M}\times\frac{\partial\vec{M}}{\partial t} \right]-\frac{\gamma H_{s}}{M_{S}}\left[ \vec{M}\times\left[ \vec{M}\times\vec{\sigma} \right] \right]-\gamma H_{s}\left[ \vec{M}\times\vec{\sigma} \right]$ (S9_1)

Herein, $\vec{\sigma}$ is the polarization vector of the spin current, *α* is the damping constant, *M*_s_ is the saturation magnetization, *γ* is the gyromagnetic ratio, $H_{s}$ is the spin-torque coefficient, which is proportional to the spin Hall angle $\alpha_{H}$, and *ζ* is the field-like coefficient, showing the ratio between FL torque and SL torque.

The effective magnetic field ($\vec{H}_{\mathrm{eff}}$) is given by:

$\vec{H}_{\mathrm{eff}}=-\frac{\delta E_{\mathrm{tot}}}{\vec{\delta M}}$; $E_{\mathrm{tot}}=\int dV\left\{ E_{\mathrm{ani}}+E_{\mathrm{ex}}+E_{\mathrm{ext}}+E_{d} \right\}$ (S9_2)

where total energy ($E_{\mathrm{tot}}$) is the summation of the anisotropy energy ($E_{\mathrm{ani}}$), exchange energy ($E_{\mathrm{ex}}$), demagnetization energy ($E_{d}$), and Zeeman energy ($E_{\mathrm{ext}}$), and $E_{\mathrm{ext}}$ = 0 because the external field is not applied in our simulation for the field-free switching manner. The equations to calculate these energies can be referred to in our previous papers [19-21].

The parameters used for the simulations are taken from our experimental data [22]: *M*_s_ = 1.4 T, with an exchange constant *A* = 10^-11^ J/m. The channel layer is our triple-domain structured (100) RuO_2_ with $\alpha_{H}$ = 0.01, and *ζ* = 6. We compared the simulation results for two devices with *ζ* = 0, and *ζ* = 6. The interfacial effects also were taken into account by considering the change of interfacial anisotropy which might lead to the change in the effective anisotropy *E*_ani_ of the device, thus changing the $\vec{H}_{\mathrm{eff}}$. Using micro-magnetic simulations, the time evolution of the magnetization (Fig. S9) can be obtained by solving the LLG equation. Based on this data, we summarize the critical switching voltage.





**Fig. S9. The time evolution of the magnetization for 75°-canted MTJ device at the write pulse of 8 ns and 3 V. The switching time (*τ*) is defined as the time at which the magnetization of the free layer is switched from the parallel state to the anti-parallel state with the reference layer.**

**S10: Exclusion of the interfacial Rashba effect:**

We prepared a control sample: RuO_2_ (4)/Ru (0.5)/Co_20_Fe_60_B_20_ (1.2)/MgO (1.3)/Ta (1.0) (hereafter, RuO_2_/Ru/CFB) where the interfacial condition was modified by adding a thin Ru layer into the stack, compared with the Ru/CFB sample. The harmonic Hall measurement was conducted for the devices fabricated on these samples. Fig. S10 shows the 1^st^ and 2^nd^ harmonic Hall resistance curves for the devices on RuO_2_/CFB and RuO_2_/Ru/CFB, where no significant change was observed for these devices. This suggested that the contribution of the interfacial Rashba effect is negligible in the RuO_2_/CFB sample.

A potential concern could be the discontinuity and/or the diffusion and oxidation of Ru leading to the formation of a Ru-O in the Ru (0.5) layer. In such a case, the interfacial conditions of the RuO_2_/Co-Fe-B and RuO_2_/Ru(O)/Co-Fe-B are also different from each other. If the observed SOT originated from the interfacial effect, we should observe some difference in the Harmonic Hall measurement. However, the results in Fig. S10 for these devices showed a negligible change, which supports the exclusion of the interfacial effect.


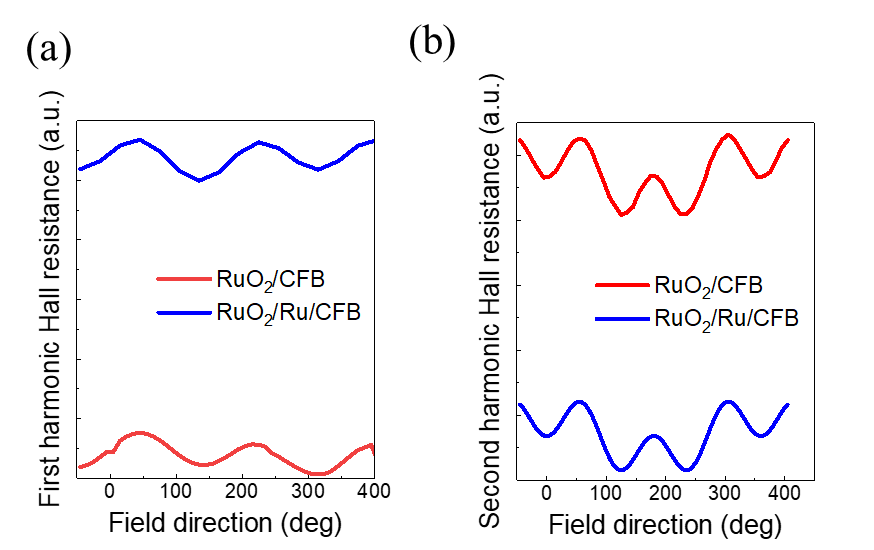


Fig. S10. First and second harmonic Hall resistance curves for RuO_2_/CFB and RuO_2_/Ru/CFB samples are shown in (a) and (b), respectively.

References:

1. T. Ichinose, H. Naganuma, Magnetic and ferroelectric properties of oxygen octahedron/tetrahedron mixed ultrathin multiferroic layer by oxygen desorption, Jour. Appl. Phys. 129, 034101 (2021).
2. T. Harano; G. Shibata; K. Ishigami; Y. Takashashi; V. K. Verma; V. R. Singh; T. Kadono; A. Fujimori; Y. Takeda; T. Okane; Y. Saitoh; H. Yamagami; T. Koide; H. Yamada; A. Sawa; M. Kawasaki; Y. Tokura; A. Tanaka, Role of doped Ru in coercivity-enhanced La_0.6_Sr_0.4_MnO_3_ thin film studied by x-ray magnetic circular dichroism, Appl. Phys. Lett. 102, 222404 (2013).
3. Z. Hu, H. von Lips, M. S. Golden, J. Fink, G. Kaindl, F. M. F. de Groot, S. Ebbinghaus, and A. Reller, Multiplet effects in the Ru L_2,3_ *X*-ray-absorption spectra of Ru(IV) and Ru(V) compounds, Phys. Rev. B 61, 5262 (2000).
4. D. J. Thouless, M. Kohmoto, M. P. Nightingale, M. den Nijs, Quantized Hall Conductance in a Two-Dimensional Periodic Potential, Phys. Rev. Lett. 49, 405 (1982).
5. J. Qiao, J. Zhou, Z. Yuan, W. Zhao, Calculation of intrinsic spin Hall conductivity by Wannier interpolation, Phys. Rev. B 98, 214402 (2018).
6. R. Gonzalez-Hernandez, L. Smejkal, K. Vyborny, Y. Yahagi, J. Sinova, T. Jungwirth, J. Zelezny, Efficient Electrical Spin Splitter Based on Nonrelativistic Collinear Antiferromagnetism, Phys. Rev. Lett. 126, 127701 (2021).
7. A. Bose, N. J. Schreiber, R. Jain, D-F. Shao, H. P Nair, J. Sun, X. S. Zhang, D. A. Muller, E. Y. Tsymbal, D. G. Schlom, D. C. Ralph, Tilted spin current generated by the collinear antiferromagnet ruthenium dioxide, Nat. Elec. 5, 267 (2022).
8. Y. Shiratsuchi, H. Oikawa, S. Kawahara, Y. Takechi, T. Fujita, R. Nakatani, Strong Perpendicular Magnetic Anisotropy at Co(111)/α-Cr_2_O_3_(0001) Interface, Appl. Phys. Express 5, 043004 (2012).
9. S. Fukami, T. Anekawa, C. Zhang, H. Ohno, A spin–orbit torque switching scheme with collinear magnetic easy axis and current configuration, Nat. Nanotech. 11, 621 (2016).
10. R. Itoh, Y. Takeuchi, S. DuttaGupta, S. Fukami, and H. Ohno, Stack structure and temperature dependence of spin-orbit torques in heterostructures with antiferromagnetic PtMn, Appl. Phys. Lett. 115, 242404 (2019).
11. Y. Takeuchi, C. Zhang, A. Okada, H. Sato, S. Fukami, H. Ohno, Spin-orbit torques in high-resistivity-W/CoFeB/MgO, Appl. Phys. Lett. 112, 192408 (2018).
12. S. Hasegawa, Characterization of Materials, pp.1925-1938, 2012 edited by Elton N. Kaufmann, John Wiley & Sons, Inc.
13. L. Liu, C.-F. Pai, Y. Li, H.W. Tseng, D.C. Ralph, and R.A. Buhrman, Spin-Torque Switching with the Giant Spin Hall Effect of Tantalum, Science 336, 555, 2012.
14. P. C. van Son, H. van Kempen, P. Wyder, Boundary Resistance of the Ferromagnetic-Nonferromagnetic Metal Interface, Phys. Rev. Lett. 58, 2271 (1987).
15. A. Endo, N. Hatano, H. Nakamura, R. Shirasaki, Fundamental relation between longitudinal and transverse conductivities in the quantum Hall system, J. Phys.: Condens. Matter. 21, 345803 (2009).
16. S. Roychowdhury, P. Yanda, K. Samanta, C. Yi, M. Yao, F. Orlandi, P. Manuel, D. Khalyavin, E. G. D. Valle, P. Constantinou, V. N. Strocov, M. G. Vergniory, C. Shekhar, C. Felser, Giant Room-Temperature Topological Hall Eﬀect in aSquare-Net Ferromagnet LaMn_2_Ge_2_, Adv. Mat. 36, 2305916 (2024).
17. See http://fujitsu.com/global/about/resources/news/press-releases/2015/0324-01.html for Fujitsu Ltd.
18. E. C. Stoner, E. P. Wohlfarth, Philos. Trans. R. Soc., A 240, 599 (1948).
19. H. Naganuma, H. Sato, S. Ikeda, T. Endoh, AIP Advances 10, 075106 (2020).
20. H. Naganuma, H. Honjo, C. Kaneta, K. Nishioka, S. Ikeda, T. Endoh, AIP Advances 12, 125317 (2022).
21. T. V. A. Nguyen, H. Naganuma, H. Honjo, S. Ikeda, T. Endoh, AIP Advances 14, 025018 (2024).
22. H. Honjo, T. V. A. Nguyen, T. Watanabe, T. Nasuno, C. Zhang, T. Tanigawa, S. Miura, H. Inoue, M. Niwa, T. Yoshiduka, Y. Noguchi, M. Yasuhira, A. Tamakoshi, M. Natsui, Y. Ma, H. Koike, Y. Takahashi, K. Furuya, H. Shen, S. Fukami, H. Sato, S. Ikeda, T. Hanyu, H. Ohno, T. Endoh, IEDM Tech. Dig. 28.5.1 (2019). (doi:10.1109/IEDM19573.2019.8993443)
